# Salvage radiotherapy for biochemical recurrence after radical prostatectomy: does the outcome depend on the prostate cancer characteristics?

**DOI:** 10.1590/S1677-5538.IBJU.2018.0039

**Published:** 2019-04-01

**Authors:** Gustavo Arruda Viani, Ana Carolina Hamamura, Alexandre Ciuffi Correa, Felipe Teles de Arruda

**Affiliations:** 1 Faculdade de Medicina de Ribeirão Preto da Universidade de São Paulo (FMRP-USP), Ribeirão Preto, SP, Brasil

**Keywords:** Radiotherapy, Prostatectomy, Prostatic Neoplasms

## Abstract

**Objective:**

To build a model to evaluate the impact of salvage radiotherapy (SRT) in men with PSA rise or persistent PSA after undergoing radical prostatectomy (RP).

**Materials and Methods:**

The study included 107 node-negative patients treated with SRT after RP at a single institution. Patients received SRT for either prostate-specific antigen (PSA) rising, or PSA persistence after RP. All patients received local radiation to the prostate / seminal vesicle bed. The primary measured outcome was the biochemical recurrence (BCR) free survival. Multivariable Cox regression analysis was used to develop a risk-stratification group to identify predictive factors associated with the probability of BCR at 5yr.

**Results:**

At a median follow-up of 52 months, the BCR free survival rate and overall survival in 5 years was 73% and 94%, respectively. At multivariable analysis, pre-SRT PSA level > 0.35ng / mL (p = 0.023), negative margins (p = 0.038), and seminal vesicles invasion (p = 0.001) were significantly associated with BCR free survival. Three risk groups using regression analysis for SRT administration was built. Low-, intermediate- and the high-risk groups had a BCR free survival in 5-years of 96%, 84%, and 44% (p = 0.0001), respectively.

**Conclusions:**

We developed a risk group stratification to show the impact of SRT based on prostate cancer characteristics. SRT showed to be extremely beneficial for patients with low- and intermediate-risk tumors. Moreover, the risk-group built could identify patients classified as high-risk who might benefit from more aggressive treatment for SRT.

## INTRODUCTION

The treatment of biochemical recurrence (BCR) after radical prostatectomy (RP) is a clinically significant issue for radiation oncologists. It is estimated that about 30% of patients undergoing RP develop an increase in prostate-specific antigen (PSA) after radical surgery (1). Among the available treatment options for BCR after RP, salvage radiation therapy (SRT) is considered one of the most common treatment options employed in clinical practice. Although its use is recommended, its effectiveness has been found to be profoundly dependent on the PSA level at the time of treatment (2, 3). Despite the absence of randomized controlled data, many retrospective studies have shown that early SRT (SRT) was associated with improved BCR-free survival, metastasis-free survival, and cancer-specific survival (4, 5). Based on these findings, eSRT is indicated specifically at a PSA level < 0.5ng / mL (2, 3).

Notwithstanding, the potential benefit of SRT must be weighed against the potential deleterious effect on functional outcomes, particularly, erectile function and urinary continence (6). Additionally, a considerable proportion of patients treated with post-surgery radiotherapy may experience early and late high-grade toxicity (7). BCR after RP has a natural history very well described and established, which does not often translate into clinical progression followed by cancer-related death (5, 8-10). However, the question if there are specific categories of patients in whom the effectiveness of SRT is superior or not to others remains open (11).

Consequently, patients with BCR and an indolent natural history may be overtreated with eSRT. In the opposite, ideal candidates for SRT may have the SRT postponed until the presence of occult metastatic disease at the time of PSA rise. Based on these concepts, we hypothesized that the impact of SRT on the biochemical control varies according to the clinical and pathological features of patients. Therefore, this study intended to build a model to evaluate the impact of SRT based on the clinical and pathological characteristics of men with PSA rise or persistent undergoing radical prostatectomy.

## MATERIALS AND METHODS

One hundred and seven patients treated with SRT at one tertiary referral center between 2009 and 2015 were identified. The inclusion criteria were: patients undergoing RP with histologically confirmed ≥ pT2 tumor and submitted to pelvic lymphadenectomy (limited or extended) with pN0 adenocarcinoma of the prostate. The extension of pelvic lymphadenectomy varied according to the initial patient's risk group. All patients should have a Karnofsky performance status (KPS) > 70. Patients submitted to pelvic radiotherapy or combined treatment with androgen blockage were excluded from this cohort.

SRT was delivered for either PSA rising or PSA persistence after RP. PSA persistence was determined as a serum concentration ≥ 0.1ng / mL at one month after RP (2). SRT was delivered to the prostate and seminal vesicle bed. The clinical target volume (CTV) was drawn on computed tomography images. The CTV included the prostatic bed, periprostatic tissue, and the seminal vesicle bed. Clinical findings, pre-surgery computed tomography scan, and surgical clips guided the clinicians for the CTV definition. The planned target volume (PTV) was determined as CTV plus a 0.7-1.0cm margin due to the organ motion and the setup error. All patients were treated with high-energy photon beams (6mV) with a conventional fractionation (1.8-2Gy / fraction). Both techniques (three-dimensional conformal (3D-RT) and intensity-modulated radiation therapy (IMRT)) were used during the study period. The following clinical and pathological data were collected: age, preoperative risk group, preoperative PSA level, postoperative PSA level, the time between RP and PSA failure, pre-SRT PSA level, SRT dose, treatment technique, pathologic stage, pathologic Gleason score, surgical margin status, seminal vesicle invasion and extracapsular extension. The primary endpoint of this cohort was the biochemical failure after SRT. BCR after SRT was considered as the first PSA measure > 0.2ng / dL. Overall survival was established as death from any cause. Follow-up time was defined as the time between SRT and the BCR or last follow-up. Time zero was set at the time of SRT.

The secondary outcome of the study consisted of acute and late genitourinary (GU) and gastrointestinal (GI) toxicity, graded according to the Radiation Therapy Oncology Group and European Organization for Research and Treatment of Cancer criteria.

### Statistical analysis

Statistical analysis was performed in three steps. In the first step, recognized predictive factors related with BCR found in the literature were tested in a univariate analysis using log-rank test. Variables with a p-value < 0.05 were selected to the next step. The second statistical step consisted of the Cox multivariate regression analysis with a bootstrapping correction. Bootstrapping is a method for obtaining robust estimates of standard errors and confidence intervals for estimates such as regression coefficient. Bootstrapping was adjusted to resample in 1000 samples. This statistical technique is most useful as an alternative to parametric estimates when the assumptions of those methods are in doubt as in the case of regression models with small subgroup samples. Variables with p with p < 0.05 in the Cox regression model with bootstrapping were led to the third step. The third step consisted of the building of a risk group for SRT and BCR free survival. The risk group was classified as low-, intermediate- and high -risk groups according to the presence of significant factors identified in the multivariate analysis. This group was tested for BCR free survival rate using the Kaplan Meier and log-rank test, with a p-value < 0.05 considered significant. SPSS version 23.0 was used to perform all statistical analysis.

## RESULTS

**Figure 1:**
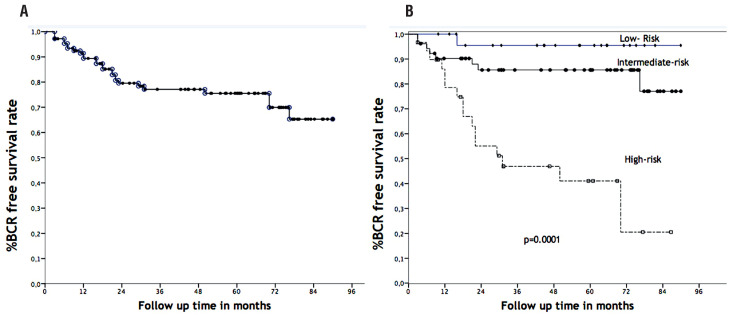
Biochemical recurrence free survival for the entire cohort (a) and biochemical recurrence for the risk groups (b).

Descriptive characteristics of patients included in this cohort are described in Table-1. Overall, 78 (73%) patients received SRT for a rising PSA, whereas 29 (27%) received SRT for PSA persistence. The median SRT dose was 70Gy (IQR; 66, 70). IMRT was used in 66% of patients and 3D RT in 34%. Time between surgery and PSA recurrence was 14 months (IQR: 7, 39). The median pre-PSA level at SRT was 0.32 (IQR: 0.23, 0.52) ng / mL. The median follow-up time from SRT was 52 months (IQR: 36, 72); during follow-up, 26 patients had a BCR after SRT and 6 deaths were observed. In 5-years, the BCR rate after SRT was 73% (Figure-1a). Univariate analysis identified six variables associated with BCR. These variables were negative margin (p = 0.003), extracapsular extension (p = 0.003), preoperative risk group (p = 0.005), seminal vesicle invasion (p = 0.001), PSA level > 0.35ng / mL (p = 0.010) at SRT, and SRT dose < 70Gy (p = 0.018) (Table-2). The Cox regression identified PSA > 0.35ng / dL (p = 0.023), negative margins (p = 0.038), and seminal vesicles invasion (p = 0.001) as independent factors related to a poor BCR free survival (Table-3).

**Table-1:** Descriptive characteristics of 107 patients undergoing radical prostatectomy (RP) with rising PSA after surgery and submitted to salvage radiotherapy (SRT).

Variables		Median (IQR)
**Age at SRT**		70 (64 – 75)
**Pre operative PSA ng/mL**		9 (6 – 13)
**Pathological stage**		
	pT2a-c	41(38.3%)
	pT3a	39(36.4%)
	pT3b	27(25.3%)
**Pathological Gleason score**		
	≤ 6	20 (18.7%)
	7	74 (69.2%)
	8-10	13 (12.1%)
**Postoperative PSA persistence**		
	No	78(72.9%)
	Yes	29(27.1%)
**Risk group pre-surgery**		
	Low	13(10.5%)
	Medium	46(43.8%)
	High	48(45.7%)
**Surgical margin status**		
	Positive	80(74.7%)
	Negative	27(25.3%)
**Time between RP and SRT (mo)**		14 (7-39)
**PSA at SRT (ng/mL)**		0.32 (0.23 – 0.52)
**RT technique**		
	3D-RT	71(66.4%)
	IMRT	35(32.7%)
**SRT dose (Gy)**		70 (66 – 70)
**Follow up time after SRT (mo)**		52 (36 – 71)
**Follow up time after RP (mo)**		

**IQR =** interquartile; mo = months, **Gy =** gray; SRT = salvage radiotherapy; **RP =** radical prostatectomy; **3D-RT =** conformational radiotherapy; **IMRT =** intensity modulated radiotherapy.

**Table-2:** Univariate analysis of factors related with biochemical recurrence (BCR) after salvage radiotherapy (SRT).

Variables		% BF control in 5 years	P Value
**Age at SRT**			0.303
	< 70 years	82%	
	≥ 70 years	67%	
**Gleason**			0.125
	≤ 7	90%	
	> 7	72%	
**Extracapsular extention**			0.003
	Yes	85%	
	No	66%	
**Seminal vesicles involvement**			0.001
	Yes	85%	
	No	59%	
**Postoperative PSA persistence**			0.967
	Yes	72%	
	No	77%	
**Risk group pre surgery**			0.005
	Low	100	
	Medium	80	
	High	66	
**Surgical margin status**			0.003
	Positive	88%	
	Negative	66%	
**Time between RP and SRT (mo)**			0.623
	< 24 months	72%	
	≥ 24 months	77%	
**PSA at SRT**			0.01
	PSA < 0.35	86%	
	PSA > 0.35	62%	
**RT technique**			0.393
	3D-RT	74%	
	IMRT	76%	
**SRT dose**			0.018
	< 70 Gy	65%	
	≥ 70 Gy	79%	

**Table-3:** Cox regression analysis with bootstrapping resample for predictive factors associated with BCR after SRT.

Predictor		HR	CI95%	P value
**Seminal Vesicles status**				0.001
	Negative	1	Ref	
	Positive	4.5	1.9-10.7	
**Surgical margin status**				0.038
	Positive	1	Ref	
	Negative	2.6	1.06– 6	
**PSA at SRT**				0.023
	PSA < 0.35	1	Ref	
	PSA ≥ 0.35	2.76	1.15-6.6	

A risk-group was built using the independent factors identified in Cox regression. Patients with no risk factors identified in Cox regression analysis were classified as low-risk, and the BCR after SRT in 5-years was 96%; while patients with one risk factor were classified as intermediate - risk and had 84% of BCR rate in 5-years and patients with two or more independent factors had 44% of BCR after SRT, p = 0.0001 (Figure-1b).

The acute GU and GI toxicity grade 2 or higher according to the RTOG criteria was 2.8% and 0, whereas the rate of late GU and GI toxicity grade 2 or higher were 11% (7.8% grade 2 and 3.7% grade 3) and 0, respectively (Table-4).

**Table-4:** Maximal acute and late gastrointestinal (GI) and genitourinary toxicity according to RTOG criteria.

Grade	Acute GU	Acute GI	Late GU	Late GI
0	64 (59.8%)	93 (86.9%)	57 (53.2%)	101 (94.4%)
1	40 (37.4%)	14 (13.1%)	38 (35.5%)	6 (5.6%)
2	3 (2.8%)	0	8 (7.4%)	0
3	0	0	4 (3.7%)	0
4	0	0	0	0

## DISCUSSION

The present cohort study hypothesized that SRT has a distinct effect on cancer control depending on clinical and pathological features. Our results validated this hypothesis, once we identified three prognostic risk groups of patients in which SRT had a different outcome.

Our findings agree with other series, which reported several of the factors associated with BCR and SRT (4, 5, 12-15) (Table-5). However, in the present analysis, we classified patients according to their clinical and pathological features. Using this strategy, we build a prognostic risk-group with significant different BCR according to their classification (Figure-1b). Indeed, we observed a significant impact of SRT in low-, and intermediate-risks, whereas the outcome of SRT did not result in a satisfactory BCR free survival rate for high-risk patients. Especially, low-risk patients had a considerably favorable 5-yr BCR of 96%. In the opposite, high-risk patients did not achieve an entirely favorable 5-yr BCR rate of 44%, suggesting that these patients need more aggressive treatment such as pelvic radiation and / or combined treatment with androgen blockage.

**Table-5:** Outcomes with salvage radiotherapy (SRT) from contemporary retrospectives studies in prostate cancer patients undergoing radical prostatectomy (RP) and PSA rise.

Author	N	PSA at SRT	RT technique	RT dose	Follow-up	BCR	Predictors response
Moreira et al. (12)	102	0.6 ng/mL	NA	66 Gy	50 mo	6yr:57 %	Surgical margins Pre-SRT PSA levels
Umezawa et al. (13)	102	0.24 ng/mL	NA	64 Gy	44 mo	4yr; 51%	Pathologic stage
Parekh et al. (14)	108	0.24 ng/mL	NA	66.4 Gy	63 mo	4yr; 45%	Pre-SRT PSA levels ADT
Lohm et al. (15)	151	0.34 ng/mL	3D-RT	66.6.Gy	82 mo	5yr: 40%	Pre- SRT PSA levels Gleason score PSADT
Present study	107	0.32 ng/mL	3D-RT/IMRT	70 Gy	53 mo	5yr: 73%	Negative margins Pathological stage Pre- SRT PSA levels

In this scenario, to investigate a more aggressive approach for patients with BCR after radical prostatectomy, the GETUG-AFU 16 randomized 743 men with a BCR after radical prostatectomy (PSA > 0.1ng / mL). The arms of randomization were salvage RT with a six-month course of ADT (goserelin) or to salvage RT alone.

The combined treatment significantly prolonged the five-year progression-free survival compared with RT alone (80 vs. 62%), but with no improvement for overall survival (96 vs. 95%) (16). Another trial conducted by the RTOG group (RTOG 9601) randomized 760 men with a detectable PSA (0.2 to 4.0ng / mL) following radical prostatectomy to RT and placebo or RT with antiandrogen therapy for 24 months (bicalutamide 150mg / day). Overall survival at 12 years was 76 percent in the bicalutamide group and 71 percent in the placebo group. Prostate cancer mortality at 12 years was reduced to 8% in the bicalutamide group (p < 0.001). The beneficial effect of bicalutamide was most evident in patients with a pre-RT PSA of ≥ 0.7ng / mL. These data from randomized clinical trials show that prostate cancer with unfavorable risk factors needs more multimodal treatments (17).

On the other hand, our risk group contests the current conviction of indication of SRT using only the PSA level for driving the decision for all patients independent of disease features. Consequently, it is possible to postpone SRT in selected patients with no compromise of the oncologic outcome. Thus, our findings revealed a clear benefit of SRT in distinct subgroups of men with either BCR or PSA persistence after RP. Other previous studies have also discussed the real necessity and when administering SRT (1, 4, 5). Notwithstanding, contrasting results have been published and the question remains unanswered. Currently, three ongoing randomized clinical trials are investigating the role of early salvage radiotherapy compared with adjuvant radiotherapy in patients with unfavorable pathological features (18-20). Adjuvant RDT is well established. These trials will try to answer questions like: how to improve the selection and avoid overtreatment for patients with PSA rising? What is the best timing to SRT?

Unfortunately, due to our sample size, we could not study the interaction among the predictive variables in this cohort. However, in a recent publication, Fossati and colleagues studied 925 patients treated by SRT due to a PSA recurrence after RP in seven institutions. In their findings, a significant relationship between cancer control with SRT and PSA level was observed (21). The chance of controlling the disease was remarkably small with a PSA level higher than 1ng / mL. Thus, SRT should be delivered at the first sign of PSA rising. Although, our analysis is completely differentiated from Fossati et al. (21), our data has the same direction of them, once the pre-PSA level at SRT < 0.35ng / mL was a strong predictor for BCR free survival. A meta-analysis evaluating the relationship between the PSA level at SRT and BCR rate also reinforced our findings. In this study, more than 5.500 patients were treated with SRT. The authors observed a 2.6% loss of BCR-free survival for each incremental of 0.1ng / mL in the PSA level at the time of SRT (22). Furthermore, a recent tumor control probability model observed that the deleterious effect of increased PSA levels at the time of SRT could never be counterbalanced by increasing the SRT (23). Consequently, international guidelines suggest delivering SRT at the first sign of BCR. However, which is the best moment for SRT administration is still debated. Our data shows that the pre-PSA level is the driver to guide the decision of indicating SRT, but other predictive factors as margin and vesicles status can help to guide the decision; mainly, when these both factors are present, SRT should be administered at low PSA level. Looking at the characteristics of our risk groups, although the interval time between RP and SRT was shorter for the high-risk group than low-risk group, the PSA level at SRT was also higher in the high-risk group. This data calls attention for the kinetics of PSA, genetic differences among the prostate cancer cells and the use of refined imaging tests like PSMA-PET during the close follow-up to select adequate treatment volume and dose to SRT at a lower PSA level for these patients.

The relationship between RT total dose and biochemical control is well known in prostate cancer patients with intact prostate gland treated with radiotherapy. This relationship has led some authors to test the hypothesis that higher doses might be beneficial even in men undergoing SRT (24). However, the clinical guidelines often suggest that at least a dose of 64-65Gy should be given (3). In our study, a dose of 70Gy was associated with a better BCR than lower doses. This finding is also in agreement with other authors. For instance, Stish et al. (25) identified that SRT with a dose of 68Gy or greater significantly reduced the risk of BCR in a large contemporary cohort. Two meta-analyses also studied this question. In these studies, a 2-2.5% improvement in recurrence-free survival for each additional Gy delivered was noted (22, 26).

Regarding late toxicity, analyzes of multiple series have found approximately 15% rates of RTOG grade 2 GI toxicity and < 5% rates of grade 3 toxicity (27). Rates of grade 2 and 3GU toxicity are reported to be approximately 10% grade 2 and 5% grade 3 in both multi- and single-institution studies (28, 29). In our study to date, the reported rate of Grade 2 late GU was 8%, with 3.7% of grade 3, and with no cases of grade 2 or 3 late GI toxicity.

Finally, this cohort has inherent limitations as a retrospective, single-institution analysis, which is similar to other observational studies. However, we tried to limit other sources of biases using a strict selection criterion to evaluate the impact of SRT in different risk groups. We decided to include only patients treated with SRT delivered to the prostate bed with no pelvic radiation or combined treatment. However, even selecting an ideal sample to build a risk group stratification for SRT, we could not examine the role of PSA kinetic and neither prostate cancer-specific survival due to 5 years follow-up. Also, we could not evaluate the role of genetic arrays or use recent refined imaging tests like PSMA-PET to select or stratify more adequately patients into different risk groups for SRT.

## CONCLUSIONS

The present study confirms the satisfactory disease control with SRT in patients with PSA rise after RP. Our data also reinforce the role of several predictive factors related to the biochemical failure in that scenario. Based on the predictive factors, we could build a risk group classification to assess the risk of BCR after SRT for PSA rise after RP.

Three different risk groups were recognized based on clinical and pathologic characteristics. The risk group classification had a satisfying performance adequately distinguishing patients with distinct outcomes. The low- and intermediate risk patients had an excellent and satisfactory result with SRT, respectively. Conversely, for the high-risk patients, SRT had a poor outcome.

These findings can be useful to identify the optimal candidates for SRT and reinforce the importance of the PSA level at the time of SRT, mainly, in the presence of other significant predictive factors. External validation of these data in a large sample combined with other refined tools such as genetic arrays and PSMA-PET is necessary to help improve the cancer control while avoiding overtreatment or undertreatment.
